# Preclinical pharmacokinetic studies and prediction of human PK profiles for Deg-AZM, a clinical-stage new transgelin agonist

**DOI:** 10.3389/fphar.2024.1423175

**Published:** 2024-08-26

**Authors:** Xiaoting Gu, Xiaohe Li, Weixue Tian, Chaoyue Zheng, Yutian Cai, Xiang Xu, Conglu Zhao, Hongting Liu, Yao Sun, Zhilin Luo, Shuwen Zhu, Honggang zhou, Xiaoyu Ai, Cheng Yang

**Affiliations:** ^1^ State Key Laboratory of Medicinal Chemical Biology, College of Pharmacy and Tianjin Key Laboratory of Molecular Drug Research, Nankai University, Tianjin, China; ^2^ The National Institutes of Pharmaceutical R&D Co., Ltd., Beijing, China; ^3^ Tianjin Key Laboratory of Molecular Drug Research, Tianjin International Joint Academy of Biomedicine, Tianjin, China

**Keywords:** Deg-AZM, nonclinical pharmacokinetics, absorption, distribution, metabolism, excretion

## Abstract

**Introduction:**

Deglycosylated azithromycin (Deg-AZM), a newly developed Class I drug with good therapeutic effects on slow transit constipation, is a small-molecule transgelin agonist that has been approved for clinical trials in 2024. The preclinical pharmacokinetic profile of Deg-AZM was investigated to support further development.

**Methods:**

A LC-MS/MS method was established and validated to detected the concentration of Deg-AZM in various biological samples. *In vivo* tests such as pharmacokinetic studies in rats and dogs, tissue distribution studies in rats, and extraction studies in rats were conducted to investigated the preclinical pharmacokinetic behaviors of Deg-AZM comprehensively. The plasma protein rate of Deg-AZM was determined by rapid equilibrium dialysis method *in vitro*. The metabolic stability and metabolite profile of Deg-AZM was assessed using pooled mice, rats, dogs, monkeys and humans microsomes *in vitro*. The PK profiles of Deg-AZM in human was predicted based on physiologically based pharmacokinetic (PBPK) models.

**Results:**

The plasma protein binding rates of Deg-AZM were lower in mice and rats, higher in dogs, and moderate in humans. The metabolic process of Deg-AZM was similar in rat and human liver microsomes. From Pharmacokinetic studies in rats and dogs, Deg-AZM was rapidly absorbed into the blood and then quickly eliminated. Plasma exposure of Deg-AZM was dose dependent with no accumulation after continuous gavage administration. In addition, there is no significant gender difference in the pharmacokinetic behavior of Deg-AZM. Deg-AZM was widely distributed in the tissues without obvious accumulation, and mainly excreted from the urinary excretion pathway. Furthermore, the pharmacokinetic profiles of Deg-AZM in humans showed dose dependency.

**Conclusion:**

The pharmacokinetic profiles of Deg-AZM was fully explored, these results could provide valuable information to support the first-in-human dosage prediction and phase I clinical design.

## Graphical Abstract

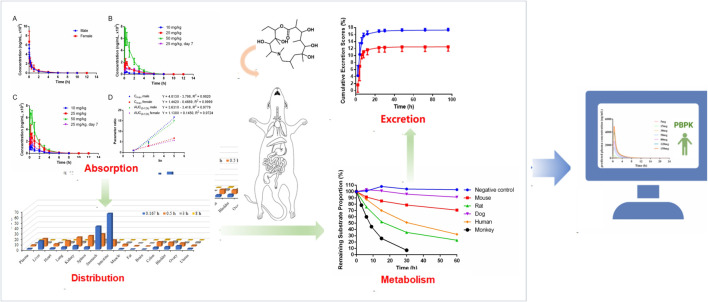

## 1 Introduction

Slow transit constipation (STC), also known as colonic inertia, is a refractory constipation caused by weakened colonic transit function, leading to the retention of fecal matter in the colon (Rao et al., 2016; Bharucha and Wald, 2019). The main clinical manifestations of STC include reduced frequency of defecation, difficulty in defecation, dry and hard feces, abdominal distension, and a series of serious complications that severely affect people’s quality of life (Bharucha et al., 2013; Shah et al., 2015). Nowadays, the incidence of constipation is increasing year by year with the acceleration of people’s pace of life, changes in dietary structure and habits, and the influence of social and physiological factors (Bharucha and Lacy, 2020; Crosby and Husk, 2021). STC is a pathological process involving multiple pathogenic mechanisms with no unified treatment plan (Pannemans et al., 2020; Vriesman et al., 2020). The first-line treatment in clinical practice is conservative drug therapy and maintaining good lifestyle habits (Camilleri et al., 2016; Yamamoto et al., 2021). Surgery, such as colon segment resection, anastomosis, and ileostomy, may be necessary in clinical practice (Huang et al., 2019). However, surgical treatment often leads to serious complications, such as recurrent constipation, diarrhea, and obstruction (Knowles, 2015). Currently, there is no ideal method for effectively treating STC, and there is an urgent need for new therapies to accelerate colonic transit. It is necessary to develop efficient and low-toxicity drugs to treat chronic constipation.

**FIGURE 1:**
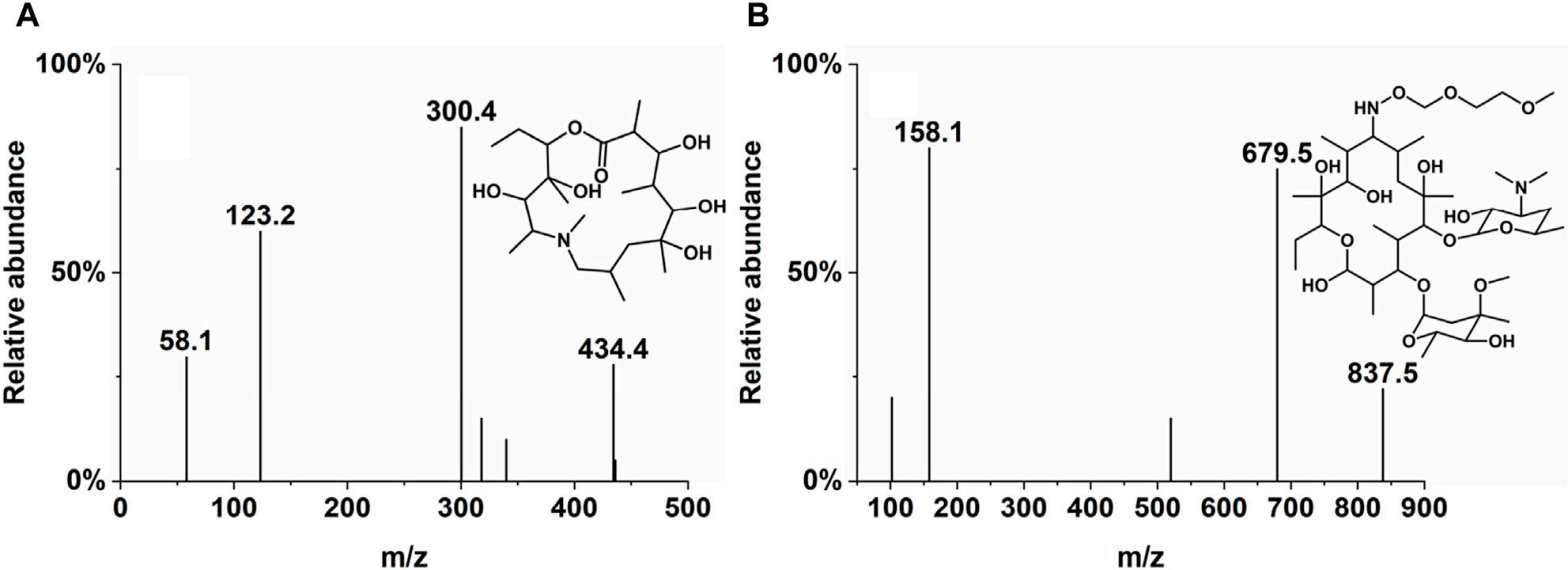
Product ion mass spectra and chemical structure of Deg-AZM **(A)** and IS **(B)**.

Deglycosylated azithromycin (Deg-AZM), a new Class I drug with good therapeutic effects on slow transit constipation developed by our research group, is a small-molecule transgelin agonist that has been approved for clinical trials in 2024. Deg-AZM is a unique metabolite of azithromycin (AZM). AZM can be used to treat gastrointestinal motility disorders (Brown et al., 1997; Parnham et al., 2014; Zeng et al., 2020). However, AZM has a side effect of gastrointestinal irritation in clinic, which varies greatly among different people (Firth and Prathapan, 2020; Ong et al., 2022; Wang et al., 2022). In addition, as an antibiotic, AZM is not suitable for development as a constipation drug. Our previous study found that Deg-AZM was a positive intestinal agonist that can induce intestinal activity, consistent with the side effect of AZM (Zhong et al., 2020). As an orally bioavailable small-molecule transgelin agonist, Deg-AZM (Figure 1) has been developed to treat STC (Zhong et al., 2020) and obtained implied approval for clinical trials from the Center for Drug Evaluation of China (acceptance number CXHL2400005). Transgelin, a 22 kDa protein, is the actin-binding protein in the Calponin family first discovered in chicken gizzard smooth muscle and named for its ability to bind to actin (Lees-Miller et al., 1987; Shapland et al., 1993; Li et al., 2008). Deg-AZM can stimulate the expression of transgelin in intestinal smooth muscle cells, promote the polymerization of G-actin into F-actin, increase the formation of stress fiber bundles in intestinal smooth muscle cells, and promote intestinal peristalsis (Zhong et al., 2020). Remarkably, because no similar drug targets transgelin to treat STC, Deg-AZM could potentially be a new option for treating STC.

Clinical pharmacokinetic (PK) data could not be obtained in the early stage of drug development. First-in-human (FIH) dosage prediction based on preclinical data is an important reference for phase I clinical design, which can effectively reduce the risk of human toxicity (Heller et al., 2018; Davies et al., 2020). Physiologically based pharmacokinetic (PBPK) modeling is a simulation model based on knowledge of physiology, biochemistry, and anatomy that simulates the blood flow in the body’s circulatory system, interconnecting various tissues or organs within the body (Kuepfer et al., 2016). It follows the principle of mass balance to simulate the metabolic processes of drugs in the body. A PBPK model could predict the PK profiles of drugs in humans (Zhang et al., 2024). The purpose of the present study was to investigate the preclinical PK profile of Deg-AZM, including the absorption, distribution, metabolism, and excretion properties of Deg-AZM across various biological matrices in nonclinical species, with a primary focus on rats and dogs. Furthermore, the PK profiles of Deg-AZM in healthy Asian humans at various doses were simulated to support the single ascending dose (SAD) clinical trial.

## 2 Materials and methods

### 2.1 Chemicals, reagents, and materials

Deg-AZM was provided by Nankai University (Tianjin, China). Roxithromycin (internal standard, IS), propranolol hydrochloride, and diphenhydramine hydrochloride were provided by the National Institutes for Food and Drug Control (Beijing, China). Midazolam was obtained from Cerilliant Co. Ltd. (Texas, United States). All of the reference substances were 98% purity. HPLC-grade methanol and acetonitrile were obtained from Thermo Fisher Scientific India Pvt. Ltd. (Waltham, MA, United States). HPLC-grade ammonium acetate was purchased from Tianjin Guangfu Technology Development Co., Ltd. (Tianjin, China). Carboxymethylcellulose sodium (CMC-Na) was provided by Sinopharm Chemical Reagent Co. Ltd. (Shanghai, China). N,N-dimethylformamide (DMF) was obtained from Concord Technology Co. Ltd. (Tianjin, China). Magnesium chloride, disodium hydrogen phosphate, and sodium dihydrogen phosphate were purchased from Tianjin Guangfu Fine Chemical Research Institute (Tianjin, China). NADP and glucose-6-phosphate dehydrogenase (G-6-PD) were purchased from Sigma-Aldrich Pty Ltd. (City of Saint Louis, United States). Blank human plasma, and liver microsomes of human, dog, mouse, and monkey were purchased from the Research Institute for Liver Diseases Co. Ltd. (Shanghai, China). Blank mouse plasma, blank rat plasma, and blank dog plasma were prepared by Tiancheng New Drug Evaluation Co. Ltd. (Tianjin, China). Deionized water used throughout the study was provided by Wahaha Corporation (Hangzhou, China).

### 2.2 Animals

Sprague–Dawley (SD) rats were purchased from Beijing Vital River Laboratory Animal Technology Co., Ltd. During the experiment, the temperature of the animal feeding environment was 19.84–26.14°C, and the humidity was 36.05–60.50%, which met the temperature and humidity requirements of the feeding environment. The beagle dogs were provided by the Shanghai Xingang Experimental Animal Research Center. During the experiment, the temperature of the animal feeding environment was 21.81–25.28°C, and the humidity was 41.94–56.39%, which met the temperature and humidity requirements of the feeding environment.

The animal experiments in this study were conducted in animal experimental facilities certified by the International Laboratory Animal Evaluation and Certification Management Committee and approved by the Institutional Animal Care and Use Committee at Tianjin Tiancheng New Drug Evaluation Co., Ltd. (Permit No. SYXK 2016–0009).

### 2.3 LC-MS/MS conditions

The concentrations of Deg-AZM in biological samples processed by precipitation of protein were analyzed by LC-MS/MS. The mobile phases used were methanol (A) and water/methanol (100:5, *v/v*) with 5 mM ammonium acetate (B). The chromatographic separation was performed on an LC-20A HPLC system (Shimadzu, Japan) using Agilent ZORBAX Eclipse C_18_ (50 mm × 4.6 mm, 5 µm). The column temperature was 40°C, and the flow rate was 0.5 mL/min. The gradient started with 55% A, progressed to 95% A over 3 min, and was maintained for 1.5 min. The column was then re-equilibrated with 55% A for 1.5 min. The mass spectrometric detection was performed on an API 4000 QTRAP (AB SCIEX) under the positive electrospray ionization (ESI+) source condition. The multiple reaction monitoring (MRM) of Deg-AZM and IS were *m/z* 434.4→300.4 and *m/z* 837.5→679.5, respectively, and are presented in Table 1 and Figure 1.

**TABLE 1:** Summary of SRM transitions used for LC-MS/MS analysis of Deg-AZM and IS.

Compound	Ion transition	CE(v)	DP (V)	EP (V)	CXP (V)
Deg-AZM	434.4→300.4	40	70	10	16
IS	837.5→679.5	32	70	10	16

### 2.4 Drug preparation before administration

An amount of Deg-AZM was weighed, dispersed in 0.5% carboxymethyl cellulose sodium solution, and then stirred evenly to prepare the formulation for oral administration to rats and dogs in *in vivo* pharmacokinetic studies, tissue distribution studies, and excretion studies*.* An amount of Deg-AZM was weighed and dissolved into N, N-dimethylformamide-5% glucose injection (7:3; *v/v*) solution to prepare the formulation for intravenous administration for rats and dogs in pharmacokinetic studies, tissue distribution studies, and excretion studies *in vivo*. All formulations were prepared freshly before dosing.

### 2.5 Biological sample preparation for LC-MS/MS analysis

#### 2.5.1 Rat plasma sample preparation

Plasma samples were prepared by protein precipitation. Aliquots (30 μL of rat plasma, 50 µL of IS solution (250 ng/mL, dissolved in acetonitrile), and 300 µL acetonitrile) were added to EP tubes. The mixtures were vortexed for 1 min and centrifuged at 12,000 rpm, 4°C for 10 min, and 50 μL of the supernatant was taken and diluted with 100 µL of distilled water, vortexed for 1 min, and centrifuged at 12,000 rpm, 4°C for 5 min. Finally, the upper layer was analyzed by the LC-MS/MS system.

#### 2.5.2 Dog plasma sample preparation

Plasma samples were prepared by protein precipitation. Aliquots (30 μL of dog plasma, 50 µL of IS solution (1 μg/mL, dissolved in acetonitrile), and 300 µL acetonitrile) were added to EP tubes. The mixtures were vortexed for 1 min and centrifuged at 12,000 rpm, 4°C for 10 min, and 50 μL of the supernatant was taken and diluted with 200 µL methanol-water (1:3, *v/v*), vortexed for 1 min, and centrifuged at 12,000 rpm, 4°C for 5 min. Finally, the upper layer was analyzed by the LC-MS/MS system.

#### 2.5.3 Tissue sample preparation

Aliquots (30 µL of the tissue homogenate, 50 µL of IS solution (250 ng/mL, dissolved in acetonitrile), and 300 µL acetonitrile) were mixed and vortexed for 1 min. After centrifuging at 4°C, 12,000 rpm for 10 min, 50 µL of the supernatant was diluted with 100 µL methanol–water (1:4, *v/v*), vortexed for 1 min, and centrifuged at 12,000 rpm, 4°C for 5 min. Finally, the upper layer was analyzed using the LC-MS/MS system.

#### 2.5.4 Urine sample preparation

Aliquots (30 µL of urine, 50 µL of 50% methanol aqueous solution, 50 µL of IS solution (1,500 ng/mL, dissolved in acetonitrile), and 300 µL of acetonitrile) were added to EP tubes. The mixtures were vortexed for 1 min, centrifuged at 12,000 rpm, 4°C for 10 min, and 50 μL of the supernatant was taken and diluted with 200 µL methanol–water (1:3, *v/v*), vortexed for 1 min, and centrifuged at 12,000 rpm, 4°C for 5 min. Finally, the upper layer was analyzed by the LC-MS/MS system.

#### 2.5.5 Feces sample preparation

Aliquots (50 µL of feces homogenate, 50 µL of 50% methanol aqueous solution, 50 µL of IS solution (250 ng/mL, dissolved in acetonitrile), and 100 µL of acetonitrile) were mixed and vortexed for 1 min. After centrifuging at 4°C, 12,000 rpm for 10 min, 100 µL of the supernatant was diluted with 100 µL distilled water, vortexed for 1 min, and centrifuged at 12,000 rpm, 4°C for 5 min. Finally, the upper layer was analyzed using the LC-MS/MS system.

#### 2.5.6 Bile sample preparation

A 50-µL aliquot of rat bile, 50 µL of 50% methanol aqueous solution, 50 µL of IS solution (250 ng/mL, dissolved in acetonitrile), and 1 mL of extraction solvent (ethyl acetate: isopropanol = 3:1, *v/v*) were added to tubes and vortexed for 1 min. After centrifuging at 4°C, 12,000 rpm for 10 min, 800 μL of the supernatant was dried with nitrogen in a 40°C water bath. The residues were reconstituted with 400 µL of 50% methanol aqueous solution, then vortexed for 1 min, and centrifuged at 12,000 rpm, 4°C for 5 min. Finally, the upper layer was analyzed using the LC-MS/MS system.

### 2.6 Method validation

The developed LC-MS/MS method for absolute quantification of Deg-AZM in biological samples was validated in accordance with the guidelines outlined in the Food and Drug Administration (FDA) Guidance for Industry on Bioanalytical Method Validation (FDA, 2018), addressing selectivity, crosstalk, carryover effect, matrix effect, recovery, linearity, lower limit of quantification (LLOQ), upper limit of quantification (ULOQ), accuracy, precision, and stability.

### 2.7 Pharmacokinetic studies in rats and dogs

Deg-AZM was administered to SD rats via a single intravenous bolus at the dose of 10 mg/kg or by single and multiple oral administration at the dose levels defined in each study. The dosages for a single oral administration were 10 mg/kg, 25 mg/kg, and 50 mg/kg, and the dose of repeated oral administration was 25 mg/kg. Six rats (half male and half female) were used for administration and sampling in each pharmacokinetic study at different doses. Blood samples (approximately 200 µL) were collected at pre-dose (0) and at 0.0333 h, 0.0833 h, 0.25 h, 0.5 h, 1 h, 2 h, 4 h, 6 h, 8 h, 10 h, and 12 h after intravenous administration. Blood samples were also collected at pre-dose (0) and at 0.0833 h, 0.25 h, 0.5 h, 1 h, 2 h, 4 h, 6 h, 8 h, 10 h, and 12 h after administration. Plasma was prepared by centrifugation at 12,000 rpm for 4 min and stored frozen at approximately −70°C until analysis.

Deg-AZM was administered to beagle dogs via a single intravenous bolus at a dose of 3 mg/kg or single and multiple oral administration at the dose levels defined in each study. The dosages for a single administration were 3 mg/kg, 10 mg/kg, and 30 mg/kg, and the dose of repeated administration by gavage administration was 10 mg/kg. Six dogs (half male and half female) were used for administration and sampling in each pharmacokinetic study at different doses. Approximately 0.5 of mL blood samples was collected at pre-dose (0) and at 0.0333 h, 0.0833 h, 0.25 h, 0.5 h, 1 h, 2 h, 4 h, 6 h, 8 h, 10 h, and 12 h post dose. Plasma was prepared by centrifugation at 12,000 rpm for 4 min and stored frozen at approximately −70°C until analysis.

Non-compartmental PK parameters were evaluated via the individual concentration–time data using WinNonlin 8.3.1 (Certara United States, Princeton, NJ), including time of maximum concentration (T_max_), maximum concentration (C_max_), area under the concentration *versus* time curve (AUC), mean residence time (MRT), volume of distribution (V*z*), and terminal half-life (T_1/2_). Finally, the plasma kinetic characteristics of the drug in rats were analyzed, including exposure level, absorption characteristics, dose relationship, elimination rate, possible accumulation, and absolute bioavailability. The T-test was used to compare data between different groups, and *P* < 0.05 was considered statistically significant. The absolute bioavailability was calculated using the average AUC value without statistical analysis.

### 2.8 Tissue distribution studies in rats

SD rats were randomly divided into four groups (tissues were taken at 0.167 h, 0.5 h, 3 h, and 8 h post dose), with six rats in each group (half male and half female). The rats were fasted for 12 h and allowed ad libitum access to water before the experiment. After oral administration of 25 mg/kg of Deg-AZM, the rats were anesthetized at different time points, and the plasma was prepared by venous blood collected from the abdominal aorta. The rats were euthanized, and brain, muscles, fat, testes (ovaries), epididymis (uterus), bladder, spleen, kidneys, liver, heart, lungs, colon, stomach, and duodenum tissues were collected. Tissue homogenates were prepared using a 50% methanol aqueous solution in a ratio of 1:5 (g/mL). The above biological samples were stored at −70°C for determination.

### 2.9 Plasma protein binding

The rapid equilibrium dialysis method was used to determine the plasma protein rates of Deg-AZM in the plasma of mice, rats, dogs, and humans *in vitro*. Propranolol was selected as the positive control drug for this study. A 0.04-mL aliquot of Deg-AZM acetonitrile solution at concentrations of 50 μg/mL, 200 μg/mL, and 1,000 μg/mL was added into 1.96 mL blank plasma of mice, rats, dogs, and humans, respectively, then mixed evenly to prepare drug-containing plasma samples with final concentrations of 1 μg/mL, 4 μg/mL, and 20 μg/mL for Deg-AZM. Then, 0.024 mL of propranolol methanol solution (2.5 μg/mL) was added into 1.176 mL blank plasma of mice, rats, dogs, and humans, respectively, with mixing evenly to prepare drug-containing plasma samples with final concentrations of 50 ng/mL for propranolol.

In the balanced dialysis tube (n = 3), drug-containing plasma (300 µL) of Deg-AZM at concentrations of 1 μg/mL, 4 μg/mL, and 20 μg/mL or propranolol at concentrations of 50 ng/mL were added to side A, respectively, and 500 µL of phosphate-buffered saline (PBS) was added to side B. The dialysis devices were sealed with a sealing film and incubated in a 37°C water bath with gentle shaking at 100 rpm for 4 h. A 30-μL sample of plasma from side A and another 30-μL sample of the solution from side B were aspirated and analyzed to determine the concentration of Deg-AZM using LC-MS/MS. The protein binding rate was calculated according to the following formula:

$$\text{Bound }\left(\%\right)=\left(C_{\text{total}}‐C_{\text{free}}\right)/C_{\text{total}}\times 100\%.$$

C_total_ represents the drug concentration on the plasma side, and C_free_ represents the drug concentration on the PBS side.

### 2.10 *In vitro* metabolic stability

The metabolic stability and metabolite profile of Deg-AZM (1 µM) were assessed using pooled mouse, rat, dog, monkey, and human microsomes (Research Institute for Liver Diseases Co. Ltd., Shanghai, China). Midazolam was employed as the positive control drug to monitor the activity of enzymes in the test. An inactivated rat liver microsome incubation system was set as the negative control group to investigate the self-stability of Deg-AZM in the incubation system.

The total incubation volume of 1 mL included 745 µL PBS (pH 7.4), 50 µL microsomal protein (final concentration at 1 mg/mL), NADPH regeneration system, and 5 µL Deg-AZM (final concentration at 1 µM). Incubation was carried out in a water bath at 37°C. At 0 min, 3 min, 6 min, 9 min, 15 min, and 30 min (for monkey microsomes) and 0 min, 6 min, 15 min, 30 min, 60 min, and 120 min (for the other four species and the negative control), 50 µL of the reaction mixture was withdrawn and added into an equal volume of cold acetonitrile to stop the reaction and precipitate proteins. A positive control group was set up to ensure the reliability of the study. At the end of the assay, the remaining content of Deg-AZM was determined by LC-MS/MS.

The elimination rate constant (−*k*e) was the slope of the linear regression of ln (% remaining content of Deg-AZM) with incubation time. T_1/2_, intrinsic clearance (CL_int_), hepatic clearance (CL_h_), and extraction ratio (ER) of Deg-AZM were calculated according to the following formula:

$$T_{1/2}=\frac{0.693}{ke}.$$

$${\text{CL}}_{\mathrm{int}}=ke/C_{\text{protein}}\times \text{SF}.$$

$${\text{CL}}_{h}=\frac{Q\cdot f_{ub}\cdot {CL}_{\mathrm{int}}}{Q+f_{ub}\cdot {CL}_{\mathrm{int}}.}$$

$$\text{ER}=\frac{{\text{CL}}_{h}.}{Q}$$

In the formula, C_protein_ was 1 mg/mL. The scaling factor (SF) = microsomal weight per gram of liver (mg/g) × liver weight to body weight ratio (g/kg). The microsomal weights per gram of the livers of mice, rats, dogs, monkeys, and humans were 45 mg/g, 44.8 mg/g, 77.9 mg/g, 45 mg/g, and 48.8 mg/g, respectively (Yang et al., 2022). The liver weight to body weight ratios of mice, rats, dogs, monkeys, and humans were 87.5 g/kg, 40.0 g/kg, 32.0 g/kg, 32.5 g/kg, and 25.7 g/kg, respectively (Yang et al., 2022; Pearson and Wienkers, 2008). SF values in mice, rats, dogs, monkeys, and humans were 3937.5 mg/kg, 1792 mg/kg, 2492.8 mg/kg, 1462.5 mg/kg, and 1254.16 mg/kg, respectively. The liver blood flow rates (Q) in mice, rats, dogs, monkeys, and humans were 90 mL/min/kg, 55.2 mL/min/kg, 30.9 mL/min/kg, 44 mL/min/kg, and 20.7 mL/min/kg, respectively. *f*_µb_ was approximately equal to 1.

### 2.11 Metabolite profiling

After incubating Deg-AZM with liver microsomes, the resulting samples from “*2.10 In vitro metabolic stability”* were analyzed by ultra-high performance liquid chromatography with quadrupole time-of-flight mass spectrometry (UPLC-QTOF-MS). UNIFI software was used to identify the metabolites of Deg-AZM. Potential metabolites were identified by combining standard metabolic reaction patterns. Based on the precise molecular weight and formula of each metabolite, fragment ions of the parent compound and metabolites were compared to elucidate the structures of the metabolites. The metabolic spectrum differences of Deg-AZM in liver microsomes of different species were analyzed by semi-quantitative peak area normalization.

### 2.12 Excretion studies in rats

Twelve SD rats (half male and half female) were used to collect urine, fecal, and bile samples after administration of Deg-AZM. Two rats of the same gender were placed in a metabolic cage. After oral administration of Deg-AZM at 25 mg/kg, urine samples were collected at 0–2 h, 2–4 h, 4–6 h, 6–8 h, 8–12 h, 12–24 h, 24–30 h, 30–48 h, 48–72 h, and 72–96 h post dose at room temperature. Fecal samples were collected at 0–6 h, 6–12 h, 12–24 h, 24–30 h, 30–48 h, 48–72 h, and 72–96 h post dose at room temperature. Six SD rats (half male and half female) were used to collect bile. Bile samples were collected from 0–2 h, 2–4 h, 4–6 h, 6–8 h, 8–12 h, 12–24 h, 24–30 h, 30–48 h, and 48–72 h post dose using bile duct intubation surgery. The volume of urine and bile and the weight of feces were measured. The above biological samples were stored at −70°C until further determination.

### 2.13 Prediction of PK profiles of Deg-AZM in healthy Asian humans based on PBPK models

#### 2.13.1 Parameters employed for the development of PBPK

Parameters related to physiological and drug-specific were collected to establish the PBPK models of Deg-AZM, including molecular weight, solubility, LogP, pKa, fraction unbound in plasma (*f*_up_), apparent permeability coefficient (P_app_), clearance in microsomes (CL), and T_1/2_. The solubility of Deg-AZM was determined in a phosphate buffer liquid system at pH 7.4. The logP of Deg-AZM was measured using a shake flask method with n-octanol/buffer. The pKa of Deg-AZM was determined by UV–visible spectrophotometry according to the *Chinese Pharmacopoeia*. The *f*_up_ of Deg-AZM could be calculated from the *“2.9. Plasma protein binding”* experiment using the following formula: *f*_up_ = 1 − (the plasma protein binding rates of Deg-AZM). The P_app_ of Deg-AZM was determined on Caco-2 cell monolayers. The CL and T_1/2_ of Deg-AZM were measured following the experiment described in *“*2.10. *In vitro metabolic stability”*.

#### 2.13.2 Establishment and validation of PBPK models for Deg-AZM in rats

PBPK models of Deg-AZM in rats were developed using PK-Sim (Version 9.1.2, Bayer Technology Services, Leverkusen, Germany). The cellular permeability of the models was estimated using the PK-Sim standard method. The Rodgers and Rowland method was employed to optimize the distribution part of the models. The simulated PK parameters of Deg-AZM in rats were compared with the observed values from experiments to validate the PBPK models. The accepted criterion was that the ratio between the predicted and observed values should fall within the range of 0.5–2.0.

#### 2.13.3 Prediction of PK profiles of Deg-AZM in healthy Asian humans

After successfully establishing and validating the PBPK models of Deg-AZM in rats, the PK profiles in healthy Asian humans of Deg-AZM at various doses were simulated to support the SAD clinical trial.

## 3 Results

### 3.1 Absorption

#### 3.1.1 Deg-AZM plasma pharmacokinetics in rats

**FIGURE 2:**
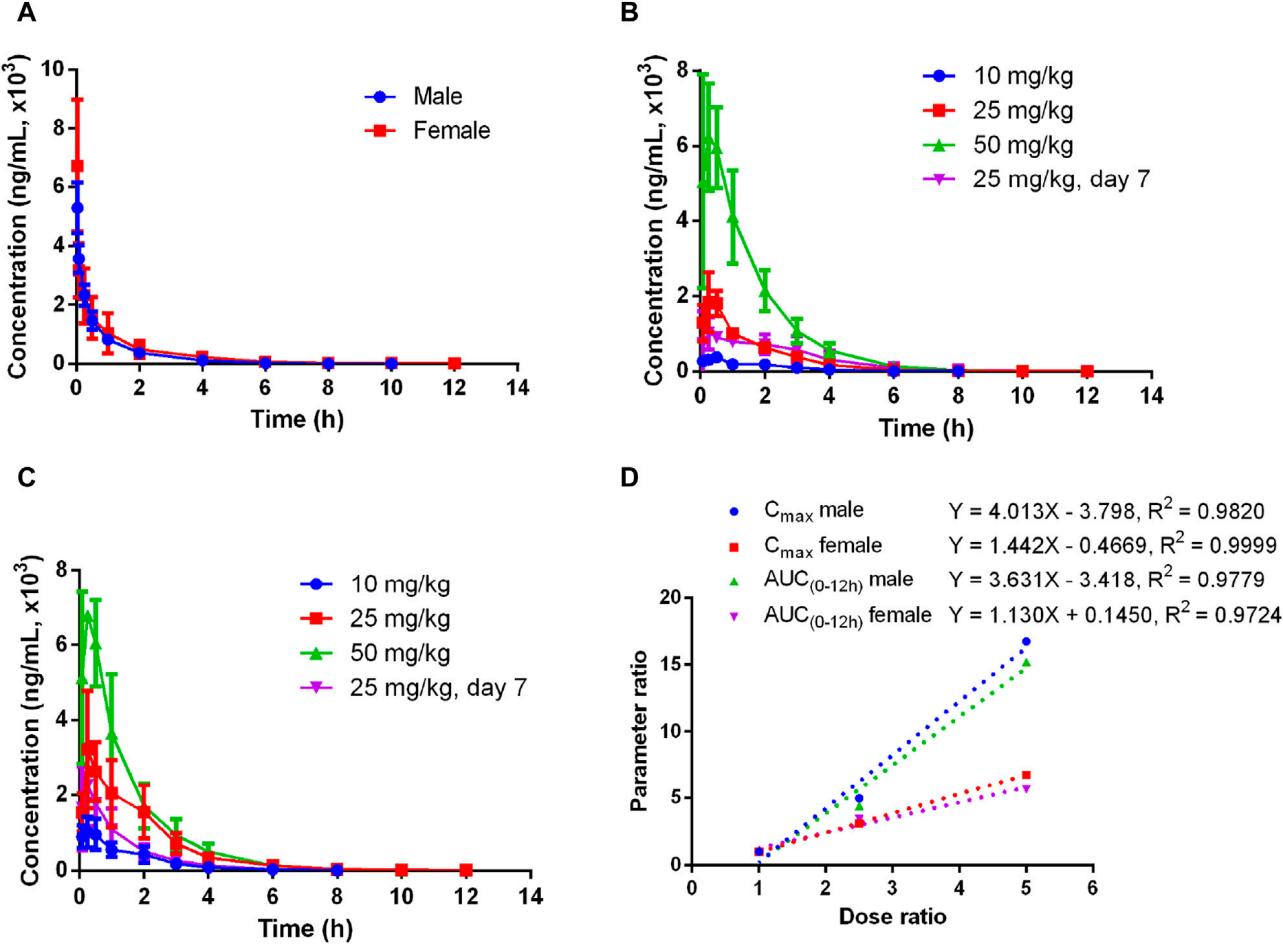
Mean plasma concentration–time curves of Deg-AZM in rats **(A)** after a single 10 mg/kg intravenous administration, **(B)** after single or multiple oral doses in male rats, **(C)** after single or multiple oral doses in female rats, and **(D)** exposure–dose relationship after single oral doses of 10 mg/kg, 25 mg/kg, and 50 mg/kg (n = 3).

The plasma pharmacokinetics of Deg-AZM in male and female rats were estimated after a single intravenous dose or single and multiple oral administration. The PK parameters are listed in Table 2. The plasma concentration *versus* time curves are displayed in Figure 2. After a single intravenous injection of Deg-AZM at a dose of 10 mg/kg, Deg-AZM was eliminated quickly with a T_1/2_ of 1.05 h and 1.72 h for male and female rats, respectively. The apparent distribution volumes (V_d_) for male and female rats were 4.85 L/kg and 6.82 L/kg, which is significantly greater than the total body fluid volume (0.6 L/kg), indicating that Deg-AZM was mainly distributed in tissues. The clearance (CL) values of Deg-AZM were 3.22 L/h/kg and 2.81 L/h/kg for male and female rats. The absolute bioavailabilities of Deg-AZM at the dosage of 10 mg/kg were 23.8% and 47.46% for male and female rats. The absolute bioavailability of Deg-AZM may increase with increased dose because the exposure increased more than the dose. There were no apparent sex differences in the PK parameters in rats. After single gavage of Deg-AZM at doses of 10 mg/kg, 25 mg/kg, and 50 mg/kg, C_max_ values were 402 ng/mL, 2000 ng/mL, and 6,730 ng/mL for males and 1,062 ng/mL, 3,287 ng/mL, and 7,117 ng/mL for females. The AUC_0–12 h_ values were 765 h⋅ng/mL, 3,367 h⋅ng/mL, and 11,633 h⋅ng/mL for males and 1927 h⋅ng/mL, 6,580 h⋅ng/mL, and 10,857 h⋅ng/mL for females, respectively. We found that C_max_ and AUC_0–12 h_ increased with increased dose, with ratios of 1:4.98:16.74 (male) and 1:3.1:6.76 (female) for C_max_ and 1:4.4:15.21 (male) and 1:3.41:5.63 (female) for AUC_0–12 h_ (Figure 2D). The T_max_ in male and female rats was 0.278–0.917 h and 0.197–0.333 h, respectively, and the absorption rate of Deg-AZM were eliminated fast both in female and male rats. The T_1/2_ values in male and female rates were approximately 1.07–1.27 h and 1.13–1.18 h, respectively, with the fast elimination rate. T_max_ and T_1/2_ did not change with dose. Compared with a single gavage administration, the C_max_ ratios of the last dose to the first dose for male and female rats were 0.58 and 0.69, and the AUC_0–12 h_ ratios were 0.98 (male) and 0.50 (female), respectively. The T_max_ and T_1/2_ remained unchanged after multiple doses compared to those after a single dose. Therefore, there was no accumulation of the drug, and there was no change in the elimination behavior.

**TABLE 2:** Overall mean Deg-AZM plasma pharmacokinetic parameters in rats after intravenous bolus or oral administration.

PK parameters	IV-10 mg/kg		PO-10 mg/kg		PO-25 mg/kg		PO-50 mg/kg		PO-25 mg/kg (day 7)	
	Male	Female	Male	Female	Male	Female	Male	Female	Male	Female
C_max_ (ng/mL)	5,287 ± 859	6,733 ± 900	402 ± 127*	1,062 ± 330	2000 ± 516	3,287 ± 1,461	6,730 ± 1715	7,117 ± 245	1,169 ± 494	2270 ± 1,086
T_max_ (h)	0.0333	0.0333	0.917 ± 0.95	0.194 ± 0.1	0.333 ± 0.14	0.333 ± 0.14	0.278 ± 0.21	0.278 ± 0.21	0.861 ± 1.01	0.25
AUC_0–12 h_ (h·ng/mL)	3,217 ± 729	4,060 ± 2018	765 ± 84	1927 ± 778	3,367 ± 429	6,580 ± 2788	11,633 ± 2901	10,857 ± 3,202	3,310 ± 1,321	3,293 ± 1,281
AUC_0-∞_ (h·ng/mL)	3,227 ± 729	4,080 ± 2018	780 ± 94	1940 ± 774	3,383 ± 434	6,607 ± 2808	11,667 ± 2902	10,867 ± 3,188	3,333 ± 1,344	3,323 ± 1,270
T_1/2_ (h)	1.05 ± 0.09	1.72 ± 0.77	1.27 ± 0.19	1.15 ± 0.12	1.18 ± 0.18	1.36 ± 0.39	1.07 ± 0.04	1.13 ± 0.04	1.34 ± 0.2	1.56 ± 0.29
MRT_0–12 h_ (h)	1.05 ± 0.14	1.44 ± 0.41	1.78 ± 0.09	1.61 ± 0.08	1.63 ± 0.26	1.83 ± 0.17	1.51 ± 0.05	1.45 ± 0.16	NA	NA
MRT_0-∞_ (h)	1.09 ± 0.13	1.52 ± 0.48	1.93 ± 0.09 *	1.68 ± 0.09	1.68 ± 0.25	1.87 ± 0.18	1.52 ± 0.05	1.47 ± 0.14	2.34 ± 0.66	1.81 ± 0.54
V_d_ (L/kg)	4.85 ± 1.01	6.82 ± 4.32	11.9 ± 2.00	9.48 ± 0.71	12.8 ± 3.16	7.8 ± 1.92	6.89 ± 1.49	7.96 ± 2.15	NA	NA
CL (L/h/kg)	3.22 ± 0.81	2.81 ± 1.08	13 ± 1.44 *	5.69 ± 2.06	7.48 ± 1.02	4.29 ± 1.86	4.48 ± 1.16	4.87 ± 1.38	NA	NA
F (%)	NA	NA	23.8	47.46	82.8	64.8	72.3	53.5	NA	NA

Data are expressed as mean ± SD (n = 3).

* *P* < 0.05, Difference between male and female rats.

#### 3.1.2 Deg-AZM plasma pharmacokinetics in dogs

**FIGURE 3:**
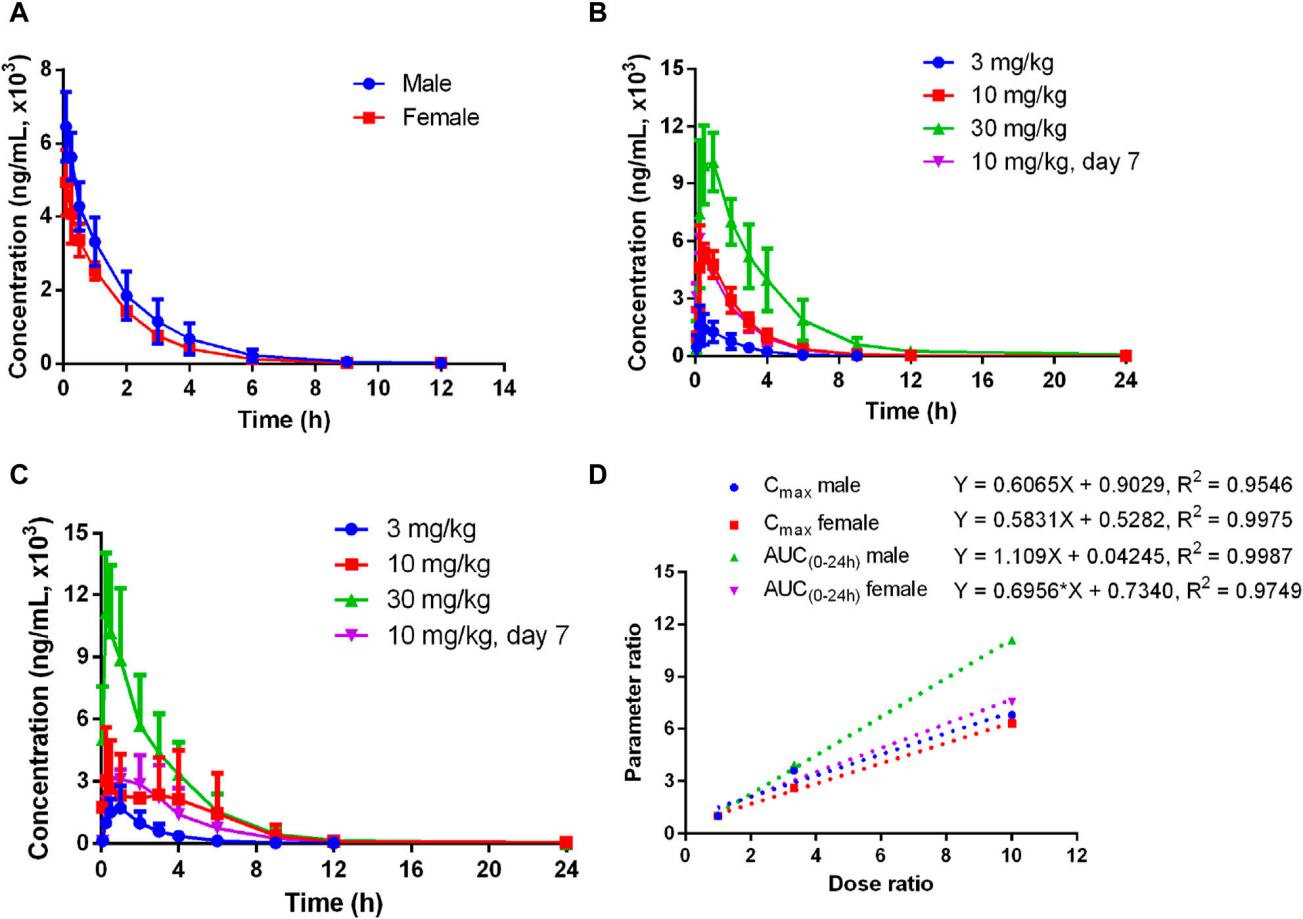
Mean plasma concentration–time curves of Deg-AZM in dogs **(A)** after a single 3 mg/kg intravenous administration, **(B)** after single or multiple oral doses in male dogs, **(C)** after single or multiple oral doses in female dogs, and **(D)** exposure–dose relationship after single oral doses of 3 mg/kg, 10 mg/kg, and 30 mg/kg (n = 3).

The plasma pharmacokinetics of Deg-AZM in male and female dogs were estimated after a single intravenous dose or single and multiple oral administration. The PK parameters are listed in Table 3. The plasma concentration *versus* time curves are displayed in Figure 3. After a single intravenous injection of Deg-AZM at a dose of 3 mg/kg in dogs, Deg-AZM was eliminated quickly with a T_1/2_ of 1.29 h and 1.36 h for male and female dogs. The absolute bioavailability of Deg-AZM at the dosage of 3 mg/kg was 32.8% and 57.4% for male and female dogs. The absolute bioavailability of Deg-AZM showed no significant change with increasing doses. There were no significant sex differences in the main PK parameters in dogs. After single gavage of Deg-AZM at doses of 3 mg/kg, 10 mg/kg, and 30 mg/kg in dogs, C_max_ were 1,617 ng/mL, 5,830 ng/mL, and 10,973 ng/mL for male dogs and 1760 ng/mL, 4,603 ng/mL, and 11,117 ng/mL for female dogs. AUC_0–24 h_ values were 3,627 h ng/mL, 14,300 h ng/mL, and 40,200 hng/mL for male dogs and 4,603 h ng/mL, 16,700 h ng/mL, and 34,700 hng/mL for female dogs, respectively. We found that C_max_ and AUC_0–24 h_ values increased with increasing dose, with a ratio of 1:3.61:6.79 (male) and 1:2.62:6.32 (female) for C_max_ and 1:3.94:11.08 (male) and 1:3.63:7.52 (female) for AUC_0–24 h_ (Figure 3D). The T_max_ in male and female rats was 0.333–0.833 h and 0.25–1.5 h, respectively, and the absorption rate of Deg-AZM was relatively fast. The T_1/2_ values in male and female dogs were 1.21–3.41 h and 1.38–2.31 h, respectively, with a faster elimination rate. T_max_ and T_1/2_ showed no significant change with increasing dose. Compared with a single gavage administration, the C_max_ ratios of the last dose to the first dose for male and female dogs were 1.05 and 0.76, and the AUC_0–24 h_ ratios were 0.96 (male) and 0.81 (female), respectively. The T_max_ and T_1/2_ remained unchanged after multiple doses compared to those after a single dose. Therefore, there was no accumulation of drugs, and there was no change in the elimination behavior.

**TABLE 3:** Overall mean Deg-AZM plasma pharmacokinetic parameters in dogs after intravenous bolus or oral administration.

PK parameters	IV-3 mg/kg		PO-3 mg/kg		PO-10 mg/kg		PO-30 mg/kg		PO-10 mg/kg (day 7)	
	Male	Female	Male	Female	Male	Female	Male	Female	Male	Female
C_max_ (ng/mL)	6,453 ± 934	4,927 ± 900	1,617 ± 1,017	1760 ± 1,035	5,830 ± 139 *	4,603 ± 270	10,973 ± 1882	11,117 ± 2911	6,123 ± 727 *	3,517 ± 766
T_max_ (h)	0.0833	0.139 ± 0.096	0.583 ± 0.38	0.583 ± 0.38	0.333 ± 0.14	1.5 ± 2.2	0.833 ± 0.29 *	0.25	0.25	1.08 ± 0.88
AUC_0–24 h_ (h·ng/mL)	11,070 ± 3,490	8,023 ± 422	3,627 ± 1880	4,603 ± 2701	14,300 ± 1,473	16,700 ± 10,078	40,200 ± 7,713	34,700 ± 15,379	13,767 ± 1,106	13,590 ± 8,239
AUC_0-∞_ (h·ng/mL)	11,093 ± 3,469	8,077 ± 419	3,657 ± 1905	4,637 ± 2707	14,400 ± 1,473	16,800 ± 10,168	40,767 ± 6,926	34,800 ± 15,379	13,833 ± 1,102	13,867 ± 8,607
T_1/2_ (h)	1.29 ± 0.13	1.36 ± 0.12	1.21 ± 0.14	1.38 ± 0.17	2.64 ± 2.04	2.04 ± 0.97	3.41 ± 0.79	2.31 ± 0.79	1.93 ± 0.47	1.56 ± 0.47
MRT_0–24 h_ (h)	1.7 ± 0.33	1.63 ± 0.18	1.87 ± 0.12	2.15 ± 0.27	2.29 ± 0.03	3.3 ± 1.99	3.59 ± 0.1	2.83 ± 0.44	NA	NA
MRT_0-∞_ (h)	1.74 ± 0.33	1.71 ± 0.2	1.93 ± 0.12	2.23 ± 0.27	2.38 ± 0.05	3.4 ± 2.07	4.03 ± 0.57 *	2.87 ± 0.43	2.25 ± 0.22	2.88 ± 1.18
V_d_ (L/kg)	0.516 ± 0.102	0.652 ± 0.1	1.77 ± 1.04	1.49 ± 0.53	2.74 ± 2.25	1.83 ± 0.22	3.79 ± 1.56	3.06 ± 1.00	NA	NA
CL (L/h/kg)	0.286 ± 0.076	0.374 ± 0.019	1.03 ± 0.62	0.779 ± 0.34	0.699 ± 0.07	0.729 ± 0.34	0.752 ± 0.14	0.989 ± 0.45	NA	NA
F (%)	NA	NA	32.8	57.4	38.8	62.4	36.3	43.3	NA	NA

Data are expressed as mean ± SD (n = 3).

* *P* < 0.05, difference between male and female rats.

### 3.2 Distribution

#### 3.2.1 Plasma protein binding

The binding rates of propranolol to plasma proteins of mice, rats, dogs, and humans were 92.7%, 90.5%, 97.0%, and 84.7%, respectively, which indicated that the experimental conditions were suitable. The *in vitro* binding rates of Deg-AZM to plasma proteins of mice, rats, dogs, and humans are shown in Table 4. The average binding rates of Deg-AZM to plasma proteins of mice, rats, dogs, and humans were 49.3%, 39.2%, 87.4%, and 73.3% in the concentration range of 1–20 μg/mL, respectively. This indicated that the plasma protein binding rate of Deg-AZM is lower in mice and rats, higher in dogs, and moderate in humans. In addition, the binding rate of Deg-AZM to human plasma proteins decreased with increasing concentration.

**TABLE 4:** The plasma protein rates of Deg-AZM in plasma of mice, rats, dogs, and humans (n = 3).

Concentration (μg/mL)	The plasma protein rates (%)			
	Mice	Rats	Dogs	Humans
1	51.1 ± 3.88	37.1 ± 1.77	93.7 ± 0.0678	86.5 ± 0.812
4	46.3 ± 4.99	39.6 ± 1.65	91.5 ± 0.378	75.4 ± 1.58
20	50.6 ± 4.06	41.0 ± 2.03	76.9 ± 1.86	57.9 ± 0.718
Average values	49.3 ± 2.63	39.2 ± 1.99	87.4 ± 9.13	73.3 ± 14.4

#### 3.2.2 Tissue distribution in rats

**FIGURE 4:**
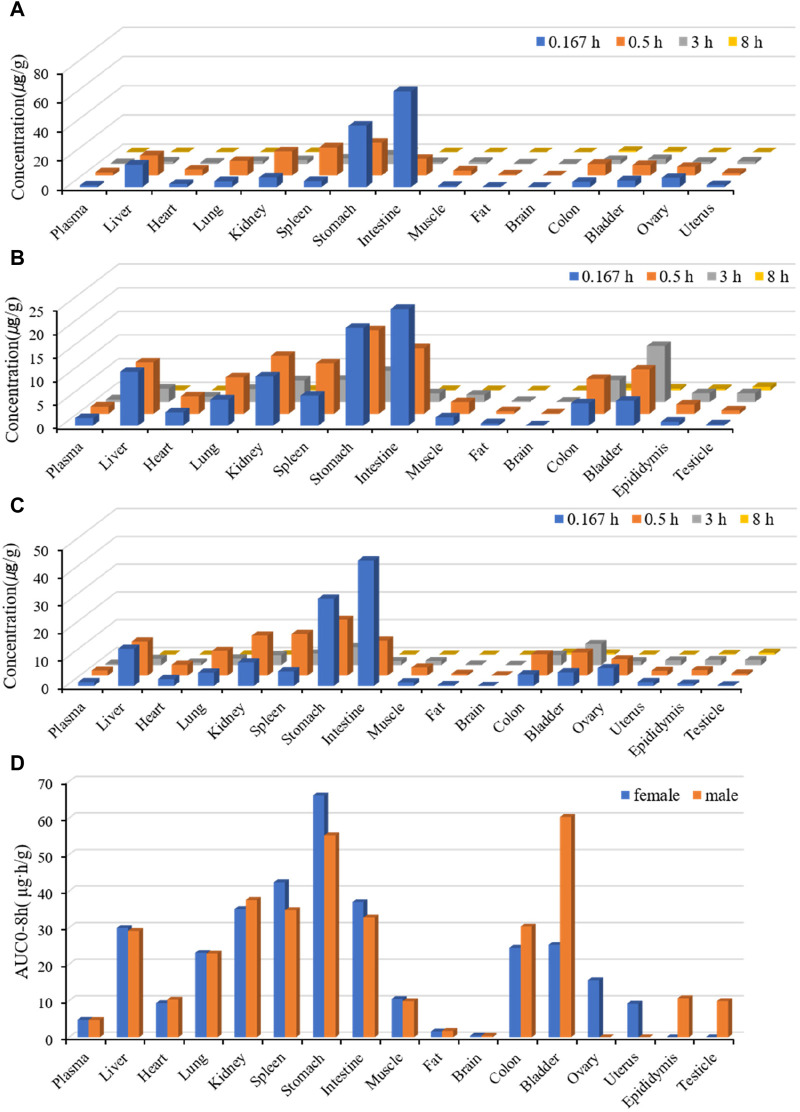
Tissue distribution of Deg-AZM at 0.167 h, 0.5 h, 3 h, and 8 h after a single 25 mg/kg oral dose. **(A)** The tissue concentration of Deg-AZM in female rats. **(B)** The tissue concentration of Deg-AZM in male rats. **(C)** The average tissue concentration of Deg-AZM in male and female rats. **(D)** The tissue AUC of Deg-AZM in male and female rats.

The tissue distributions of Deg-AZM in male and female rats are shown in Figure 4. Deg-AZM could be detected in all tissues, and the exposure level in most tissues was higher than in plasma, indicating the widespread distribution of Deg-AZM in rat tissues. There was no significant gender difference in the exposure levels of Deg-AZM in tissues (Figures 4A,B). It was consistent with the high value of V_d_ (5.83 L/kg) for Deg-AZM. The exposure levels of Deg-AZM in rat tissues were ranked as follows: stomach (12.8 times that of plasma)>bladder, spleen, kidney, duodenum, liver (6–9 times that of plasma)>colon, lungs, ovaries, epididymis, muscles, testicle, heart, uterus (2–5 times that of plasma)>plasma > fat (35.7% that of plasma)>brain (8.36% that of plasma). Deg-AZM reached peak distribution at 0.167 h in the liver, stomach, intestine, and ovaries after administration, at 3 h in testes, and at 0.5 h in other tissues. At 8 h after administration, Deg-AZM in the testicle decreased to approximately one-third of its peak distribution, while in other tissues, Deg-AZM decreased to approximately 1/100–1/10 of the peak distribution, indicating no significant accumulation of Deg-AZM in these tissues of male and female rats.

### 3.3 Metabolism

#### 3.3.1 *In Vitro* metabolic stability in liver microsomes

After incubation for 30 min in the positive control group, the remaining proportion of midazolam was 0.00338%, indicating the effectiveness of the incubation system and reaction conditions. After incubation for 120 min in the negative control group, the remaining substrate proportion was about 108%, indicating that Deg-AZM did not exhibit self-degradation in this incubation system.

**FIGURE 5:**
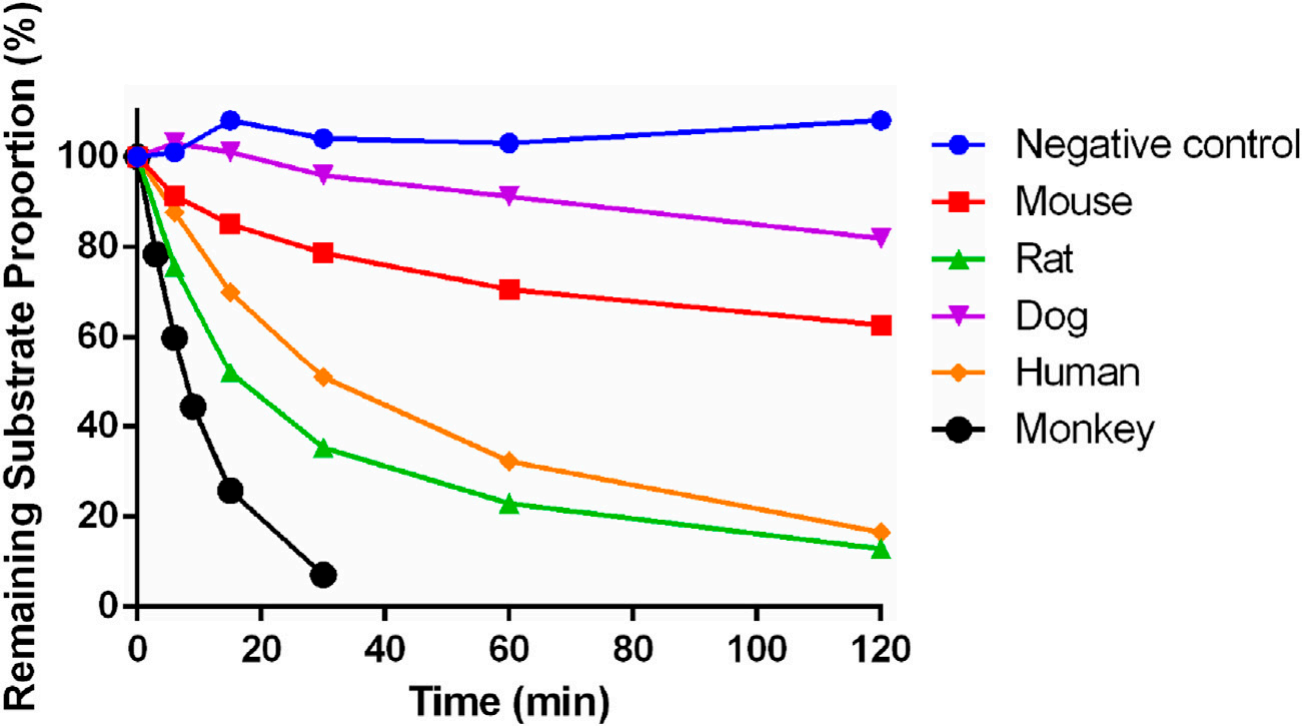
Metabolic stability of Deg-AZM in mouse, rat, dog, monkey, and human liver microsomes (n = 2).

The residual substrate ratio time curves are shown in Figure 5. After incubating Deg-AZM with mouse, rat, dog, and human liver microsomes for 120 min, the average remaining substrate ratios were 62.7%, 12.9%, 81.9%, and 16.5%, respectively. After incubating Deg-AZM with the monkey liver microsome for 30 min, the average remaining substrate ratio was 7.12%. The metabolic rate of Deg-AZM in various types of liver microsomes was in the order of monkey > rat > human > mouse > dog.

The parameters of Deg-AZM in liver microsomes are shown in Table 5. T_1/2_ was greater than 120 min, and ER was less than 0.3 of Deg-AZM in mouse and dog liver microsomes. The T_1/2_ values in rat, monkey, and human liver microsomes were between 7 min and 50 min, and the ER values were 0.343, 0.747, and 0.474 in rat, monkey, and human liver microsomes, respectively. Deg-AZM had a higher liver extraction rate in monkey liver microsome, a moderate liver extraction rate in rat and human liver microsomes, and a lower liver extraction rate in mouse and dog liver microsomes, indicating that Deg-AZM was metabolized and eliminated most rapidly in monkey liver microsomes, while it exhibited a similar metabolic elimination profile in rat and human liver microsomes.

**TABLE 5:** Parameters of Deg-AZM metabolism through various liver microsomes (n = 2).

Parameter	Mouse	Rat	Dog	Monkey	Human
Remaining proportion (%)	62.7	12.9	81.9	7.12	16.5
*k*e (min^−1^)	0.00360	0.0161	0.00180	0.0887	0.0149
T_1/2_ (min)	193	43.0	385	7.81	46.5
CL_int_ (mL/min/kg)	14.2	28.9	4.49	130	18.7
CL_h_ (mL/min/kg)	12.2	18.9	3.92	32.9	9.82
ER	0.136	0.343	0.127	0.747	0.474

#### 3.3.2 Metabolite profiling

**FIGURE 6:**
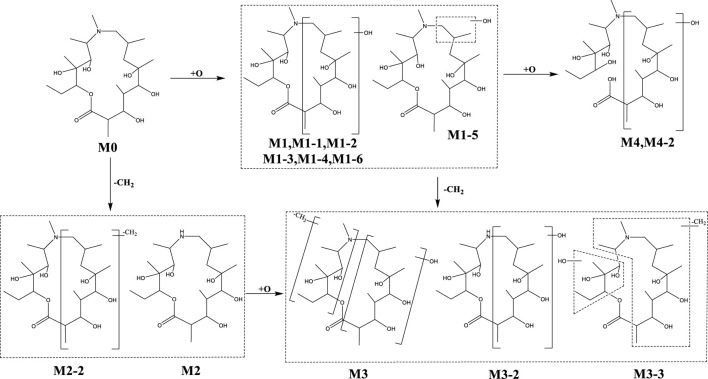
Metabolic pathway analysis of Deg-AZM in liver microsomes.

**FIGURE 7:**
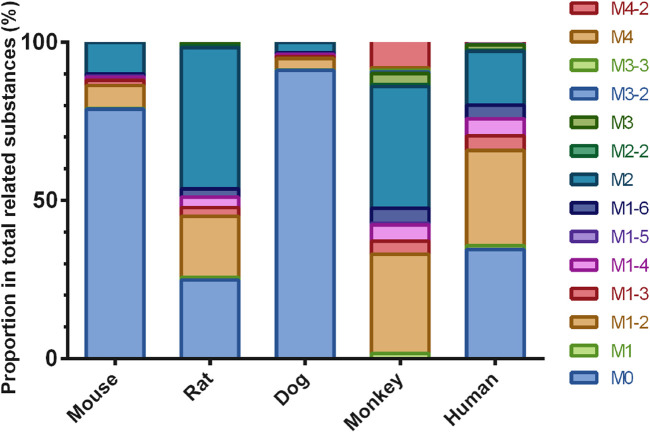
Comparison of metabolite profiles of Deg-AZM in liver microsome incubations of different species.

In addition to the prototype of Deg-AZM, 8, 8, 9, 13, and 10 metabolites of Deg-AZM were identified from the liver microsomes of mice, rats, dogs, monkeys, and humans, respectively. The metabolic pathways include hydroxylation, methylation, and combinations of different metabolic pathways. Detailed information on each metabolite is listed Table 6. The metabolic pathway analysis is shown in Figure 6. The proportion of metabolites analyzed based on the mass spectrometry response peak areas is shown in Figure 7. In the human liver microsomes, Deg-AZM, M1-2 (hydroxylation of Deg-AZM), and M2 (demethylation of Deg-AZM) were the main forms of the drug, accounting for 34.4%, 30.0%, and 17.1% of the total related substances, respectively. In the rat liver microsomes, Deg-AZM, M1-2, and M2 were the main forms of the drug, accounting for 24.8%, 19.3%, and 44.6% of the total related substances, respectively. In the mouse liver microsomes, Deg-AZM accounted for 78.8% of the total related substances, and the proportion of each metabolite was less than 10%. In the dog liver microsomes, Deg-AZM accounted for 91.1% of the total related substances, and the proportion of each metabolite was less than 4%. In the monkey liver microsomes, M1-2 and M2 accounted for 31.4% and 38.5% of the total related substances; the proportion of each metabolite was less than 9%, and Deg-AZM was not detected.

**TABLE 6:** Metabolite information of Deg-AZM identified in liver microsomes from different species.

Metabolites	Metabolic pathways	Molecular formula	*m/z* [M + H] ^+^	RT (min)	Proportion (%)				
					Mice	Rats	Dogs	Monkeys	Humans
M0	Deg-AZM	C_22_H_43_NO_7_	434.3112	7.00	78.8	24.8	91.1	—	34.4
M1	+O	C_22_H_43_NO_8_	450.3061	5.17	0.287	0.891	0.180	1.58	1.37
M1-2	+O	C_22_H_43_NO_8_	450.3061	7.32	7.27	19.3	3.56	31.4	30.0
M1-3	+O	C_22_H_43_NO_8_	450.3061	6.46	1.64	2.72	0.710	4.16	4.63
M1-4	+O	C_22_H_43_NO_8_	450.3061	8.17	0.980	3.33	0.510	5.03	5.46
M1-5	+O	C_22_H_43_NO_8_	450.3061	5.53	0.197	—	0.122	0.513	—
M1-6	+O	C_22_H_43_NO_8_	450.3061	5.35	0.834	2.63	0.389	4.76	4.18
M2	-CH_2_	C_21_H_41_NO_7_	420.2955	7.03	9.85	44.6	3.25	38.5	17.1
M2-2	-CH_2_	C_21_H_41_NO_7_	420.2955	4.99	0.126	0.590	0.0654	0.648	0.332
M3	-CH_2_+O	C_21_H_41_NO_8_	436.2904	7.16	—	1.04	0.136	3.46	1.64
M3-2	-CH_2_+O	C_21_H_41_NO_8_	436.2904	5.15	—	—	—	0.495	0.242
M3-3	-CH_2_+O	C_21_H_41_NO_8_	436.2904	5.45	—	—	—	0.613	—
M4	+2O	C_22_H_43_NO_9_	466.3010	4.69	—	—	—	0.768	—
M4-2	+2O	C_22_H_43_NO_9_	466.3010	6.02	—	—	—	8.09	0.671

—: not detected.

### 3.4 Excretion in rats

**FIGURE 8:**
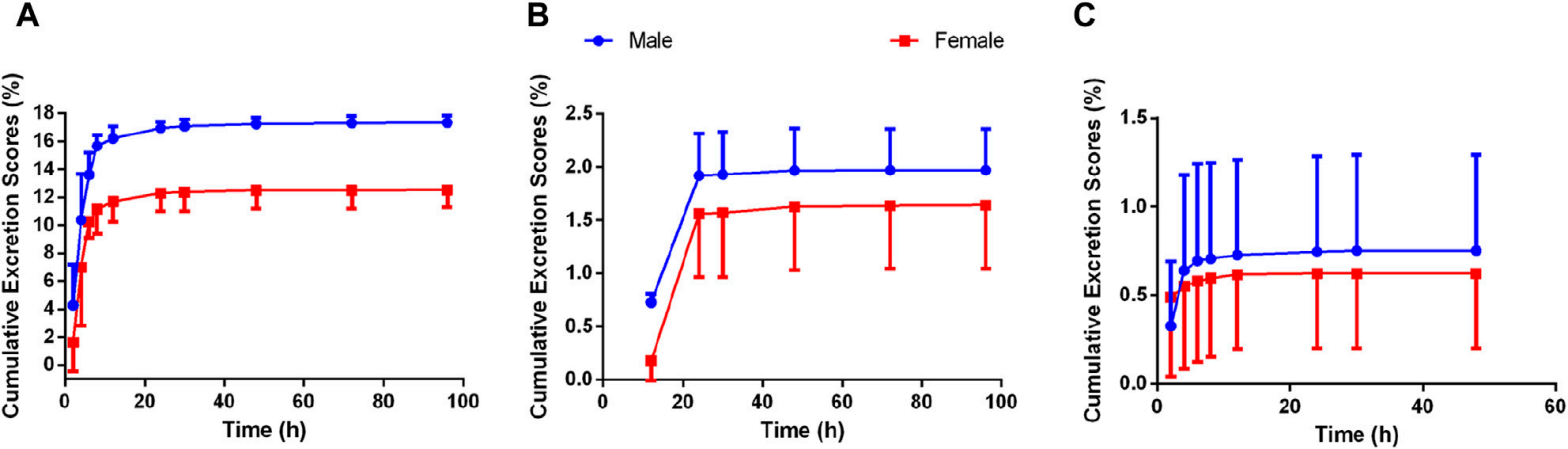
Mean cumulative excretion fraction curve of Deg-AZM in **(A)** urine, **(B)** feces, and **(C)** bile of rats after oral administration of Deg-AZM at 25 mg/kg (n = 3).

The excretion routes of Deg-AZM after oral administration of a dose of 25 mg/kg in both intact and bile duct-cannulated rats were determined. The mean cumulative excretion fraction curves of prototype Deg-AZM in the urine, bile, and feces of male and female rats are presented in Figure 8. At 96 h post-dosing, 20.022% (17.3% in urine, 0.752% in bile, and 1.97% in feces) and 14.763% (12.5% in urine, 0.623% in bile, and 1.64% in feces) of Deg-AZM were excreted from male and female rats, respectively. Deg-AZM was mainly excreted from the body through urine, with minimal excretion in its prototype form, indicating extensive metabolism within the body.

### 3.5 Establishment and validation of Deg-AZM PBPK models in rats

**FIGURE 9:**
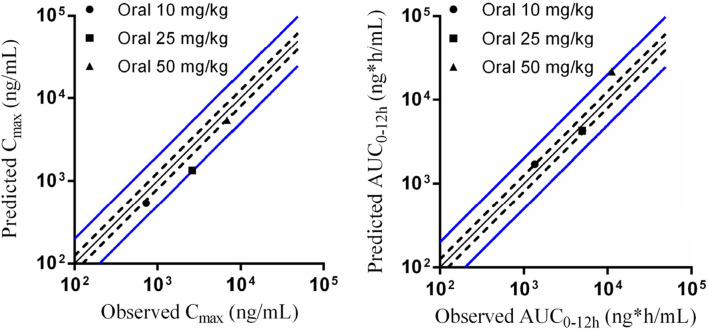
Predicted and validated PK parameters of Deg-AZM in SD rats. The solid black line represents the predicted values corresponding to the observed values. The dashed black line represents 0.8 and 1.25 times the observed values. The solid blue line represents 0.5 and 2 times the observed values.

The parameters collected from preclinical studies were used to establish PBPK models of Deg-AZM (listed in Table 7), followed by validation using PK data of rats. Figure 9 showed the predicted/observed ratios of C_max_ and AUC_0–24 h_ in rats at different oral doses, with all values falling within the acceptable range of 0.5–2.0, indicating a well-established PBPK model.

**TABLE 7:** Summary of parameters in the PBPK model.

Property (Units)	Values	Data source	Descriptions
MW (g/mol)	433.59	-	Molecular weight
LogP	1.7	Determined	Lipophilicity
pKa	9.0	Determined	Dissociation constant
Solubility (mg/L)	3,450	Determined	Solubility at pH 7.4
f_up_	0.61^a^, 0.27^b^	Determined	Fraction of free drug in plasma
P_app_ (cm/s)	3*10^−7^	Determined	Caco-2 apparent permeability
CL (mL/min/mg)	0.016^a^	Determined	Plasma clearance
T_1/2_ (min)	43^a^, 46.5^b^	Determined	T_1/2_ in microsomes
Cellular permeabilities	PK-Sim standard	Optimized	Permeability calculation method across cell
Partition coefficients	Rodgers and Rowland	Optimized	Calculation method from cell to plasma coefficient

^a^ Rat.

^b^ Human.

### 3.6 Prediction of PK profiles for Deg-AZM in a healthy Asian population

**FIGURE 10:**
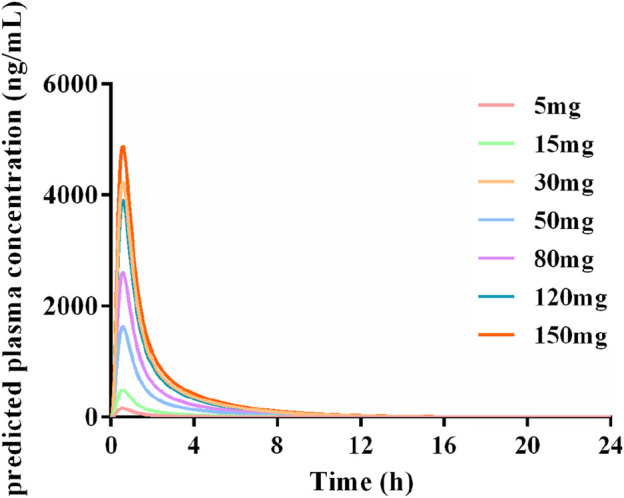
Simulated PK profiles of Deg-AZM in a healthy Asian population at doses of 5 mg, 15 mg, 30 mg, 50 mg, 80 mg, 120 mg, and 150 mg.

Figure 10 showed the PK profiles of Deg-AZM in healthy Asian individuals at seven different doses, which were simulated based on the established PBPK model. The PK behavior of Deg-AZM showed dose dependency. These results could provide valuable information for SAD studies in a FIH clinical trial.

## 4 Discussion

Deg-AZM, a new Class I drug for clinical treatment of STC, was found to stimulate the expression of transgelin in intestinal smooth muscle cells, promote the polymerization of G-actin into F-actin, increase the formation of stress fiber bundles in intestinal smooth muscle cells, and promote intestinal peristalsis (Zhong et al., 2020). Because no similar drug targets transgelin to treat STC, Deg-AZM could potentially be a new option for treating STC.

In the early stage of drug development, comprehensively elucidating the ADME properties of the drug candidate in animals is a crucial prerequisite for understanding its efficacy and safety (Lucas et al., 2019). Predicting the first human dose based on preclinical data is a crucial reference for phase I clinical trial design, as it can effectively reduce the risk of human toxicity (Heller et al., 2018; Davies et al., 2020). In this study, an LC-MS/MS method was developed and validated to quantify the concentration of Deg-AZM in various biological matrices. The ADME profiles of Deg-AZM were investigated systematically *in vivo* and *in vitro*. PK studies conducted in rats or dogs indicated no significant gender differences in the PK behavior of Deg-AZM. Additionally, Deg-AZM was rapidly absorbed into the bloodstream and then rapidly cleared from the body. The plasma exposure of Deg-AZM was dose-dependent, and there was no accumulation after continuous oral administration. Results from plasma protein binding assays showed that the plasma protein binding rate of Deg-AZM was low in mice and rats, high in dogs, and moderate in humans. Metabolic stability studies in liver microsomes revealed that Deg-AZM was more easily metabolized and eliminated in monkey liver microsomes, with similar levels of metabolism and elimination in rat and human liver microsomes. Therefore, rats and dogs were chosen as animal species in preclinical experiments. Deg-AZM was widely distributed in the tissues of rats without significant accumulation. Deg-AZM was primarily excreted from the body of rats through the urinary tract. These studies provided comprehensive preclinical data for Deg-AZM, supporting initial human dose predictions and phase I clinical trials.

PBPK modeling based on computer simulation can simulate a holistic model by building on the physiological, biochemical, anatomical, pharmacological, and thermodynamic properties of the body. The PBPK model describes the behavior of drugs *in vivo* from a mechanistic perspective (Kostewicz et al., 2014; Kaur et al., 2018). In addition, the PBPK model can also simulate the effects of metabolism, enterohepatic circulation, physicochemical parameters, transport proteins, *in vitro* data, or the influence of ingestion (Pathak et al., 2019). In recent years, PBPK models have been widely applied in drug development, including dose selection for FIH (Damle et al., 2011), drug interaction studies (Dong et al., 2022; Ni et al., 2023), drug formulation studies (Wang et al., 2009), food effects studies (Arredondo et al., 1995), as well as the PK study of special populations (such as pregnant women, children, older people, and patients with liver and kidney dysfunction) (Chetty et al., 2018; Fan et al., 2022; Pillai et al., 2022; Chen et al., 2023). Various professional computer software programs are available for PBPK modeling, such as Simcyp Simulator, GastroPlus, and PK-Sim (Wang et al., 2023). In this study, PK-Sim software was selected to establish the PBPK models of Deg-AZ-M. By inputting PK parameters, physicochemical and biological properties obtained from preclinical studies, as well as anatomical, physiological, and biochemical parameters of the human body, PBPK models of Deg-AZM in rats and dogs were established and further validated using the observed PK real data of rats and dogs. All the predicted/observed ratios of C_max_ and AUC_0–24 h_ fell within the acceptable range of 0.5–2.0, indicating a well-established PBPK model. Animal PK data can be extrapolated to humans, which, to some extent, solves the problem of ethical limitations that make it difficult to obtain certain human data. The predicted PK profiles in humans indicated that the PK behavior of Deg-AZM was dose dependent.

## 5 Conclusion

This study revealed the preclinical PK profile of Deg-AZM, a new transgelin agonist at the clinical stage. No significant gender differences were noted in the PK behavior of Deg-AZM in rats and dogs. After oral administration, Deg-AZM was rapidly absorbed into the blood, widely distributed in the tissues without obvious accumulation, and quickly eliminated from the body. Repeated administration did not change the elimination behavior of Deg-AZM. Deg-AZM was mainly excreted from the body through the urinary excretion pathway. The predicted PK profiles in humans indicated that the PK behavior of Deg-AZM was dose dependent. Clinical PK data could not be obtained in the early stage of drug development, making this study an important reference for reducing the risk of human toxicity in phase I clinical design.

## Data Availability

The raw data supporting the conclusions of this article will be made available by the authors, without undue reservation.

## Abbreviations

AUC, area under the concentration *versus* time curve; AZM, azithromycin; CL_h_, hepatic clearance; CL_int_, intrinsic clearance; C_max_, maximum concentration; CMC-Na, carboxymethylcellulose sodium; Deg-AZM, deglycosylated azithromycin; DMF, N,N-dimethylformamide; ER, extraction ratio; ESI, electrospray ionization; glucose-6-phosphate dehydrogenase; IS, internal standard; *k*e, elimination rate constant; LC-MS/MS, liquid chromatography coupled with tandem mass spectroscopy; LLOQ, lower limit of quantification; m/z, mass-to-charge ratio; MF, matrix factor; MRM, multiple reaction monitoring; NA, not applicable; NADP, nicotinamide adenine dinucleotide phosphate; PBPK, physiologically based pharmacokinetic. PK, pharmacokinetics; QC, quality control; RE, relative error; RSD, relative standard deviation; STC, slow transit constipation; t_1/2_, elimination half-life; T_1/2_, terminal half-life; T_max_, time of maximum concentration; ULOQ, upper limit of quantification; V_d_, apparent distribution volume.
